# Spatio-temporal patterns of childhood pneumonia in Bhutan: a Bayesian analysis

**DOI:** 10.1038/s41598-021-99137-8

**Published:** 2021-10-14

**Authors:** Kinley Wangdi, Kinley Penjor, Tsheten Tsheten, Chachu Tshering, Peter Gething, Darren J. Gray, Archie C. A. Clements

**Affiliations:** 1 Department of Global Health, Research School of Population Health, College of Health and Medicine, Australian National University, Canberra, Australia; 2 Vector-Borne Diseases Control Programme, Department of Public Health, Ministry of Health, Thimphu, Bhutan; 3 Royal Centre for Disease Control, Ministry of Health, Thimphu, Bhutan; 4 Child Health Program, Communicable Diseases Division, Department of Public Health, Ministry of Health, Thimphu, Bhutan; 5 Telethon Kids Institute, Nedlands, Australia; 6 Faculty of Health Sciences, Curtin University, Perth, Australia

**Keywords:** Diseases, Risk factors

## Abstract

Pneumonia is one of the top 10 diseases by morbidity in Bhutan. This study aimed to investigate the spatial and temporal trends and risk factors of childhood pneumonia in Bhutan. A multivariable Zero-inflated Poisson regression model using a Bayesian Markov chain Monte Carlo simulation was undertaken to quantify associations of age, sex, altitude, rainfall, maximum temperature and relative humidity with monthly pneumonia incidence and to identify the underlying spatial structure of the data. Overall childhood pneumonia incidence was 143.57 and 10.01 per 1000 persons over 108 months of observation in children aged < 5 years and 5–14 years, respectively. Children < 5 years or male sex were more likely to develop pneumonia than those 5–14 years and females. Each 1 °C increase in maximum temperature was associated with a 1.3% (95% (credible interval [CrI] 1.27%, 1.4%) increase in pneumonia cases. Each 10% increase in relative humidity was associated with a 1.2% (95% CrI 1.1%, 1.4%) reduction in the incidence of pneumonia. Pneumonia decreased by 0.3% (CrI 0.26%, 0.34%) every month. There was no statistical spatial clustering after accounting for the covariates. Seasonality and spatial heterogeneity can partly be explained by the association of pneumonia risk to climatic factors including maximum temperature and relative humidity.

## Introduction

Pneumonia is a major cause of morbidity and mortality^1^. Each year, pneumonia accounts for over 12 million hospital admissions and 1.3 million deaths in children aged < 5 years worldwide^2,3^. In 2017, pneumonia was the second-leading cause of death in children aged < 5 years and it is estimated to remain the case through to 2040^4^. Mortality is disproportionately higher in low-income and middle-income countries (LMICs), where 95–99% of pneumonia-specific < 5 deaths occur^5^. Children < 5 years in LMICs experience 0.28 episodes per child-year, with the highest rates and predominantly reported in South Asia and sub-Saharan Africa^6,7^.

Pneumonia is a potentially life-threatening illness with a particularly high burden in South Asia and sub-Saharan Africa^3,8,9^. It is not only a major cause of morbidity and mortality but is also associated with a substantial economic burden on healthcare systems^10,11^ and household income^12^. Pneumonia often has a complex aetiology involving multiple pathogens, including many that are transmitted person-to-person. Past time-series analyses have identified various pneumonia and influenza outcomes to be temporally seasonal, demonstrating highly consistent peaks in winter months and troughs in summer months^13,14^. Other studies have found that pneumonia admissions were highly spatially clustered^15^, driven by contact with infected people during indoor activities^16^.

**Figure 1:**
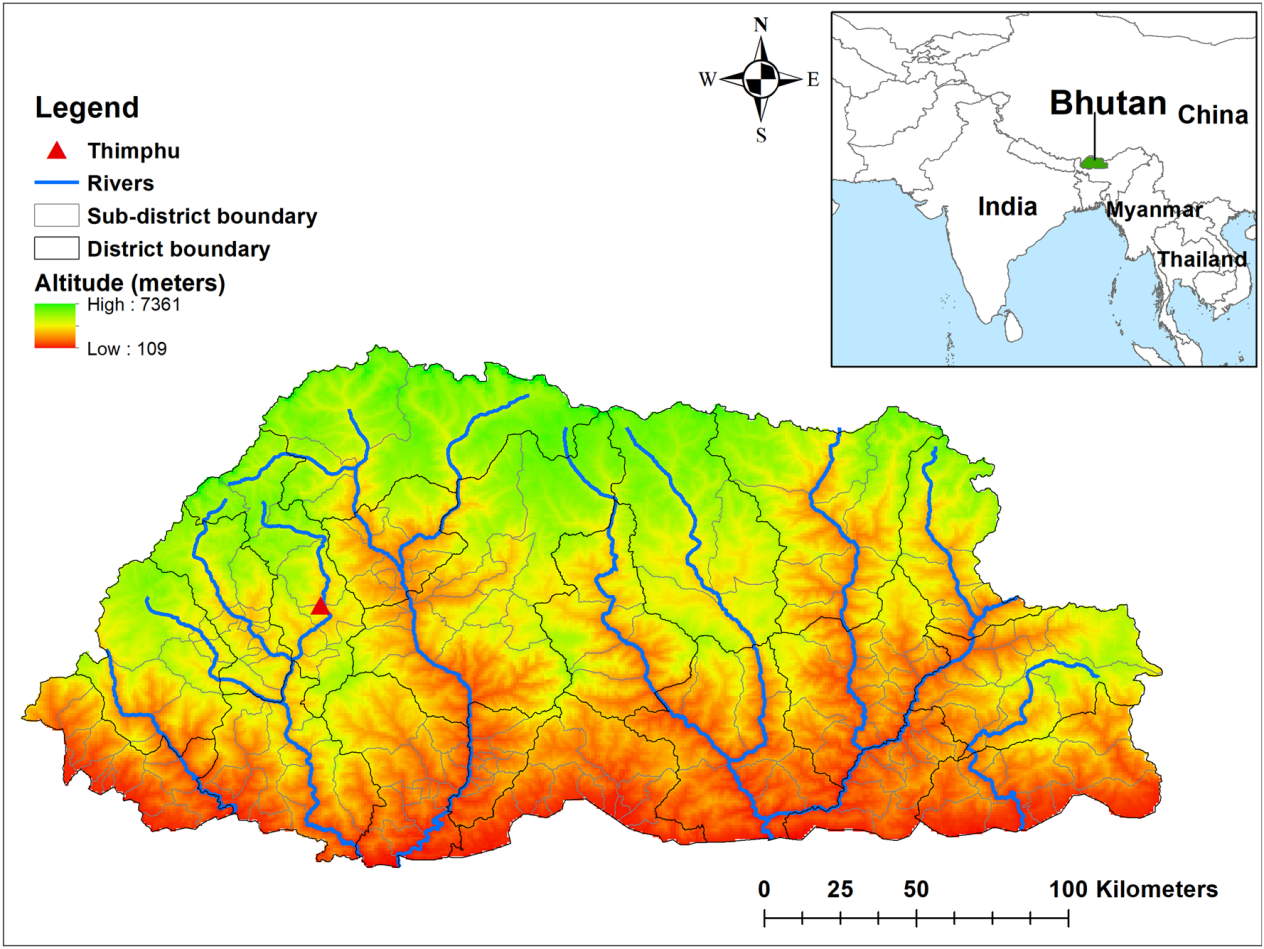
Map of Bhutan with districts and sub-districts with altitude. Map was created using ArcMap 10.5 software (ESRI, Redlands, CA).

Pneumonia continues to be an important communicable disease in Bhutan, located in the Eastern Himalayas^17–19^ (Fig. 1). In 2019, pneumonia was one of the top-ten ranked diseases in Bhutan, accounting for 19% of the overall disease burden^20^. In the financial year 2017–2018, 7.1% of the Bhutanese government’s health expenditure was spent on treating infectious respiratory diseases^19,21^. Despite the importance of pneumonia, and the infectious nature of the disease, there have been no previous studies to understand the underlying ecological drivers of pneumonia in Bhutan^22,23^. Understanding the spatial and temporal patterns of pneumonia will be important for prevention and preparedness through more efficient targeting of scarce healthcare resources. In this study, we investigated the trends of childhood pneumonia in Bhutan, identified potential high-risk geographical areas and quantified associations between disease risk and climatic risk factors.

## Results

### Descriptive analysis

**Figure 2:**
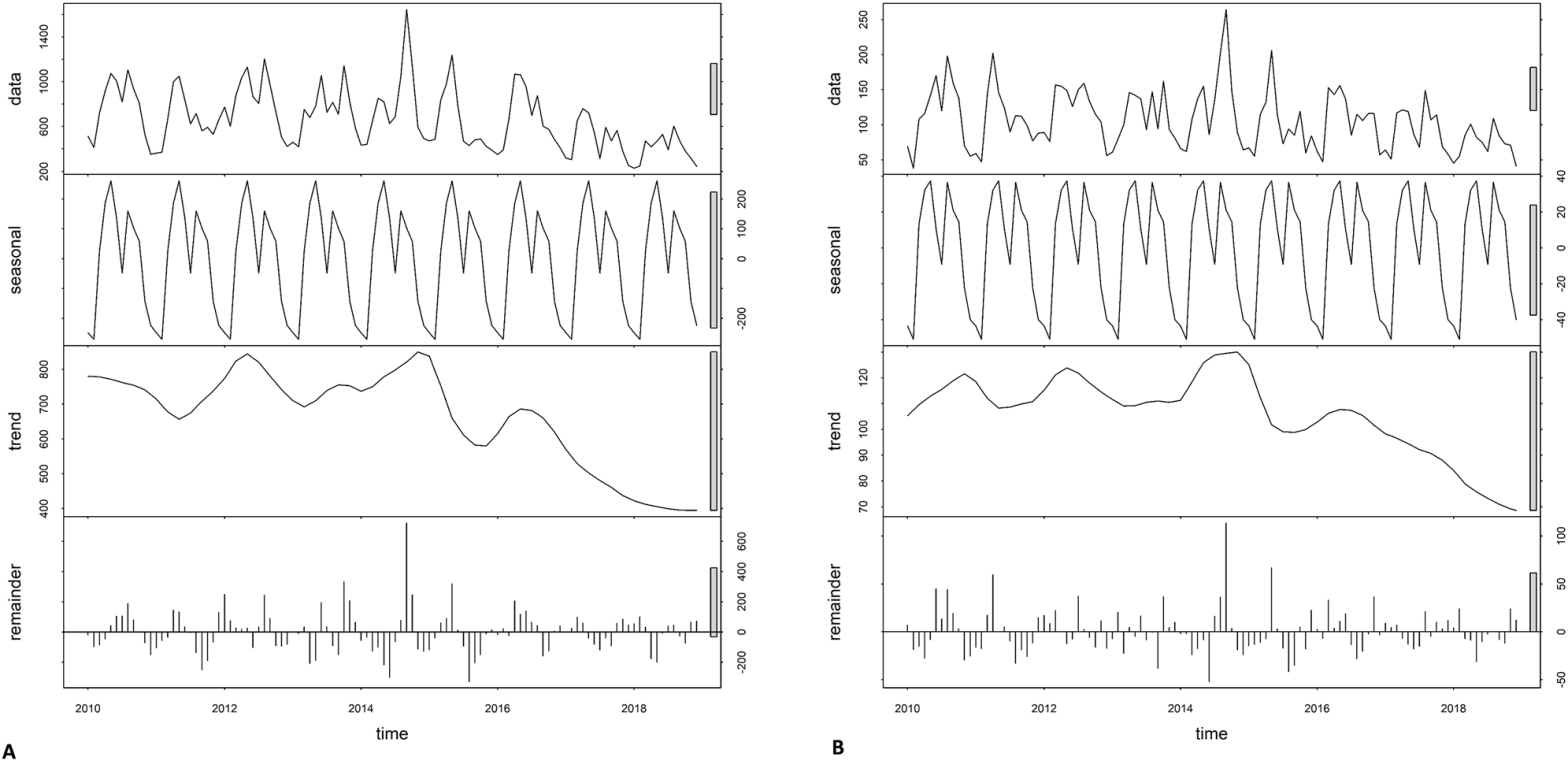
Decomposed monthly cases of pneumonia: (**a**) under 5 years and (**b**) 5–14 years during the study period, 2010–2018. (Maps were created using ArcMap 10.5 software (ESRI, Redlands, CA).

**Figure 3:**
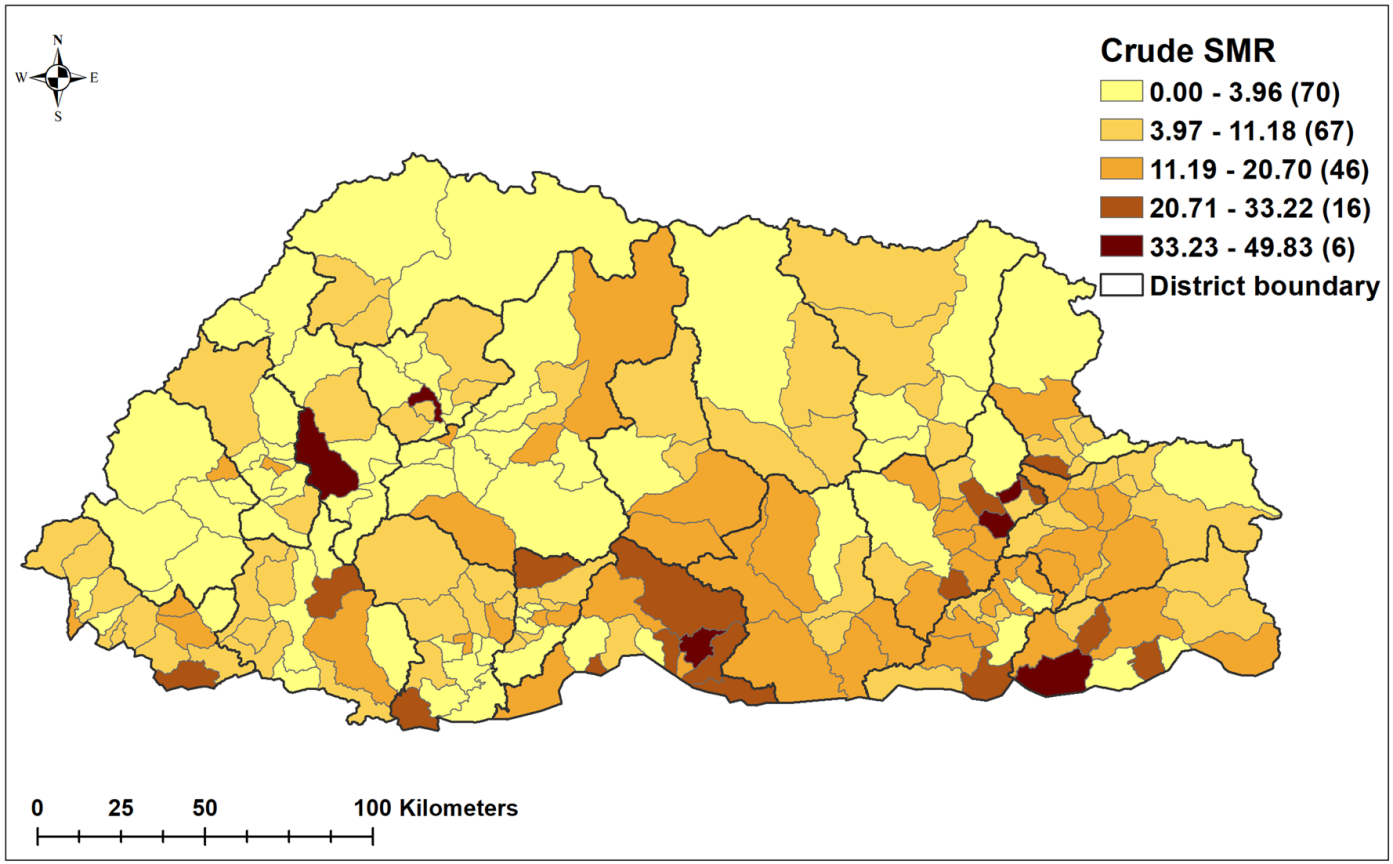
Crude standardized morbidity ratios (SMR) of pneumonia by sub-district during the study period, 2010–2018. (Maps were created using ArcMap 10.5 software (ESRI, Redlands, CA).

A total of 83,315 paediatric pneumonia cases were reported in Bhutan during the study period (2010–2018). This corresponded to 71,807 and 11,508 cases in children aged < 5 years and 5–14 years, with an incidence of 143.57 and 10.01, respectively, per 1000 persons during the nine years (Table 1). In both age groups, incidence decreased from 173.18 and 82.28 cases per 1000 persons in 2010 to 11.32 and 6.62 cases per 1000 persons in 2018 for the < 5 years and 5–14 years age groups, respectively (Table 1). The seasonal and trend decomposition using loess (STL) of monthly pneumonia cases based on locale is illustrated in Fig. 2. The highest number of cases was reported in 2012, and pneumonia displayed a strong seasonal pattern, with peaks in May and September of each year (Supplementary Fig. 1 and 2). The average standardized morbidity ratio (SMR) of pneumonia at sub-district level was 9.6 (range: 0 to 49.8; Standard Deviation of 9.7). The SMR for Haa, Paro, Gasa, Bumthang and Wandue was lower than average, whilst for Chukha, Mongar, Samtse, Sarpang, Samdrup Jongkhar and Thimphu districts, the SMR was higher than average (Fig. 3).

**Table 1:** Yearly incidence of pneumonia stratified by age.

Year	Under 5 years			5–14 years		
	Cases	Population	Incidence*	Cases	Population	Incidence*
2010	9204	53,147	173.18	1383	122,183	11.32
2011	7975	53,740	148.40	1300	123,542	10.52
2012	9939	54,337	182.91	1469	124,916	11.76
2013	8956	54,942	163.01	1336	126,305	10.58
2014	9434	55,553	169.82	1517	127,710	11.88
2015	7489	56,171	133.33	1204	129,131	9.32
2016	8150	56,795	143.50	1293	130,567	9.90
2017	5883	57,427	102.44	1123	132019	8.51
2018	4777	58,059	82.28	883	133,471	6.62

*Incidence per 1000 population.

### Spatio-temporal model

Model I, which contained the unstructured random effects, had the lowest deviation information criterion (DIC) (159.881.0), and was thus better fitting than Model II and Model III, which contained the spatially structured random effects. The incidence of pneumonia was 14 times (95% credible interval [CrI] 13.96, 14.59) higher in children aged < 5 years as compared to those 5–14 years. Females were 11.3% (95% CrI 10.0%, 12.5%) less likely to develop pneumonia compared to males. The burden of pneumonia decreased by 0.3% (95% CrI 0.26%, 0.34%) every month. Each 1 °C increase in the maximum temperature was associated with a 1.3% (95% CrI 1.27%, 1.4%) increase in pneumonia cases. Conversely, each 10% increase in relative humidity was associated with a 1.2% (95% CrI 1.1%, 12.5%) decrease in the incidence of pneumonia (Table 2).

**Table 2:** Regression coefficients, relative risk and 95% credible interval from Bayesian spatial and non-spatial models of pneumonia cases in Bhutan, January 2010-December 2018.

Model/variable	Coeff, posterior mean (95% CrI)	RR, posterior mean (95% CrI)
**Model I (unstructured)**		
α (Intercept)^†^	− 2.28 (− 2.59, − 1.32)	
Age^‡^	2.658 (2.636, 2.680)	14.268 (13.957, 14.585)
Sex^‡‡^	− 0.120 (− 0.134, − 0.105)	0.887 (0.875, 0.900)
Mean monthly trend	− 3.43 × 10^–3^ (− 4.26 × 10^–3^, − 2.58 × 10^–3^)	0.9966 (0.9957, 0.9974)
Altitude (100 m)	1.32 × 10^–5^ (− 1.84 × 10^–7^, 2.62 × 10^–5^)	1.001 (1.000, 1.003)
Rainfall (10 mm)	7.67 × 10^–4^ (− 3.96 × 10^–7^, 1.54 × 10^–3^)	1.008 (1.000, 1.015)
RH (10%)**	− 1.25 × 10^–3^ (− 1.45 × 10^–3^, − 1.06 × 10^–3^)	0.988 (0.986, 0.989)
Maximum temp (°C)	1.31 × 10^–2^ (1.26 × 10^–2^, 1.37 × 10^–2^)	1.013 (1.013, 1.014)
Extra zero^¥^	0.169 (0.162, 0.177)	
Heterogeneity		
Unstructured	1.694 (1.306, 2.149)	
Structured (trend)	0.188 (0.138, 0.238)	
DIC*	159,881.0	
**Model II (structured)**		
α (Intercept)^†^	− 2.544 (− 2.658, − 2.431)	
Age^‡^	2.659 (2.561, 2.758)	14.282 (12.949, 15.768)
Sex^‡‡^	− 0.120 (− 0.169, − 0.071)	0.887 (0.845, 0.932)
Mean monthly trend	− 3.42 × 10^–3^ (− 4.31 × 10^–3^, − 2.51 × 10^–3^)	0.9966 (0.9957, 0.9975)
Altitude (100 m)	1.34 × 10^–5^ (− 1.14 × 10^–6^, 2.77 × 10^–5^)	1.000 (1.000, 1.000)
Rainfall (10 mm)	6.80 × 10^–3^ (− 4.40 × 10^–3^, 1.81 × 10^–2^	1.007 (0.996, 1.018)
RH (10%)**	− 1.25 × 10^–3^ (− 1.54 × 10^–3^, 9.75 × 10^–4^)	0.999 (0.998, 0.999)
Maximum temp (°C)	5.93 × 10^–2^ (5.51 × 10^–2^, 6.35 × 10^–2^)	1.061 (1.057, 1.066)
Extra zero^¥^	0.169 (0.150, 0.189)	
Heterogeneity		
Structured (spatial)	1.685 (1.291, 2.147)	
Structured (trend)	0.039 (0.030, 0.048)	
DIC	160,115.0	
**Model III (mixed)**		
α (Intercept)^†^	− 2.54 (− 2.89, − 2.01)	
Age^‡^	2.568 (2.597, 2.719)	14.268 (13.423, 15.165)
Sex^‡‡^	− 0.12 (− 0.151, − 0.09)	0.887 (0.860, 0.914)
Mean monthly trend	− 3.42 × 10^–3^ (− 4.26 × 10^–3^, − 2.56 × 10^–3^)	0.9966 (0.9957, 0.9974)
Altitude (100 m)	1.33 × 10^–5^ (− 3.55 × 10^–7^, 2.68 × 10^–5^)	1.013 (1.000, 1.027)
Rainfall (10 mm)	7.64 × 10^–4^ (− 1.78 × 10^–4^, 1.70 × 10^–3^	1.008 (0.998, 1.017)
RH (10%)**	− 3.33 × 10^–2^ (− 3.78 × 10^–2^, 1.92 × 10^–2^)	0.967 (0.963, 0.981)
Maximum temp (°C)	5.93 × 10^–2^ (5.62 × 10^–2^, 6.24 × 10^–2^)	1.061 (1.058, 1.064)
Extra zero^¥^	0.169 (0.157, 0.182)	
Heterogeneity		
Unstructured	1.67 (1.29, 2.13)	
Structured (spatial)	0.32 (0.21, 0.48)	
Structured (trend)	0.08 (0.05, 0.13)	
DIC	159,983.0	

*coeff* coefficients, *CrI* credible interval, *RR* relative risk, *DIC* deviation information criterion.

*Best-fit model.

^†^Coefficient.

^‡^Reference- 5–14 years.

^‡‡^Reference-male.

**RH- relative humidity lagged 3 months.

^¥^Probability of extra zero.

**Figure 4:**
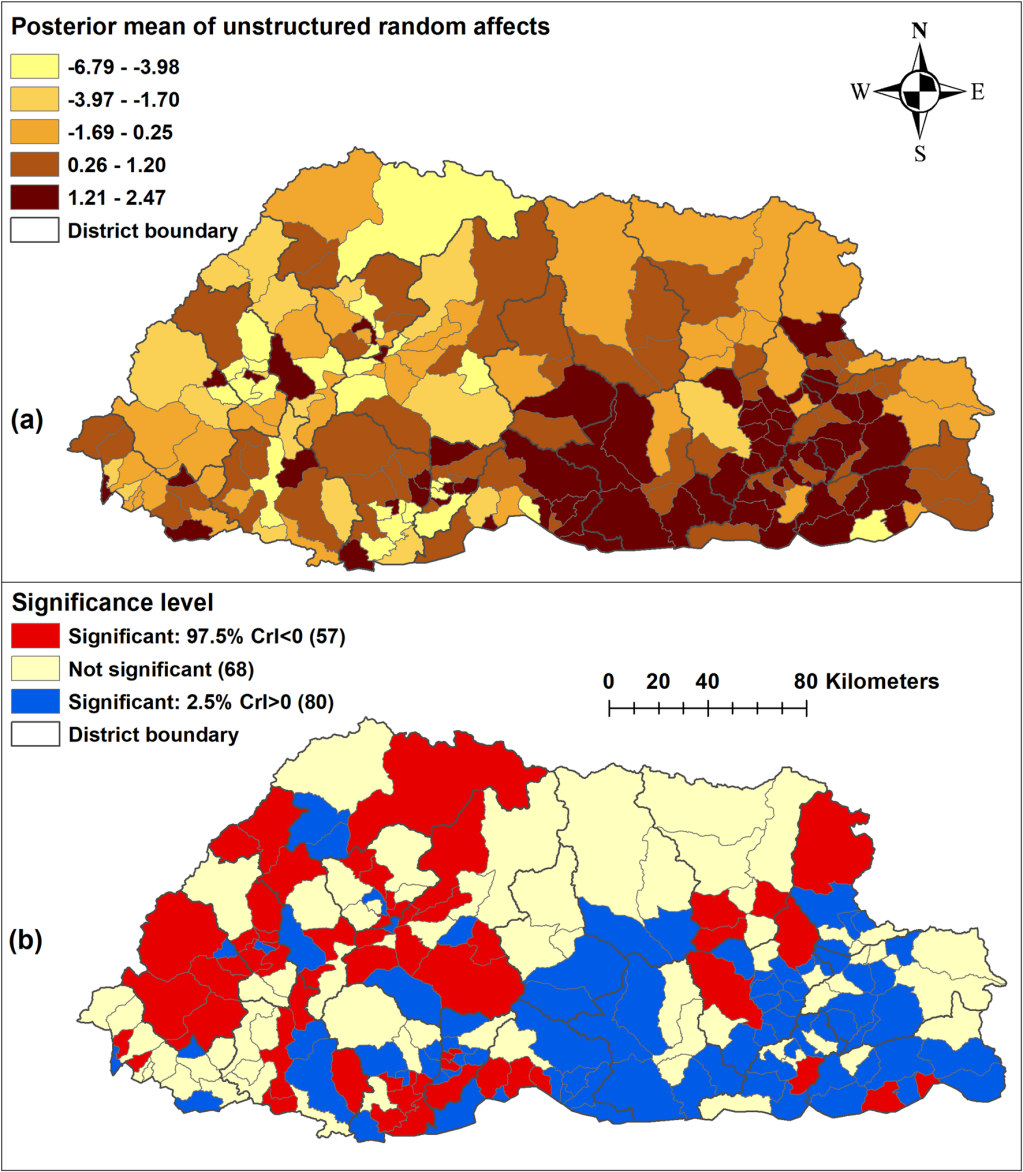
**(a)** Spatial distribution (**b**) significance map of the posterior means of unstructured random effects of pneumonia in Bhutan, 2010–2018. (Maps were created using ArcMap 10.5 software (ESRI, Redlands, CA).

**Figure 5:**
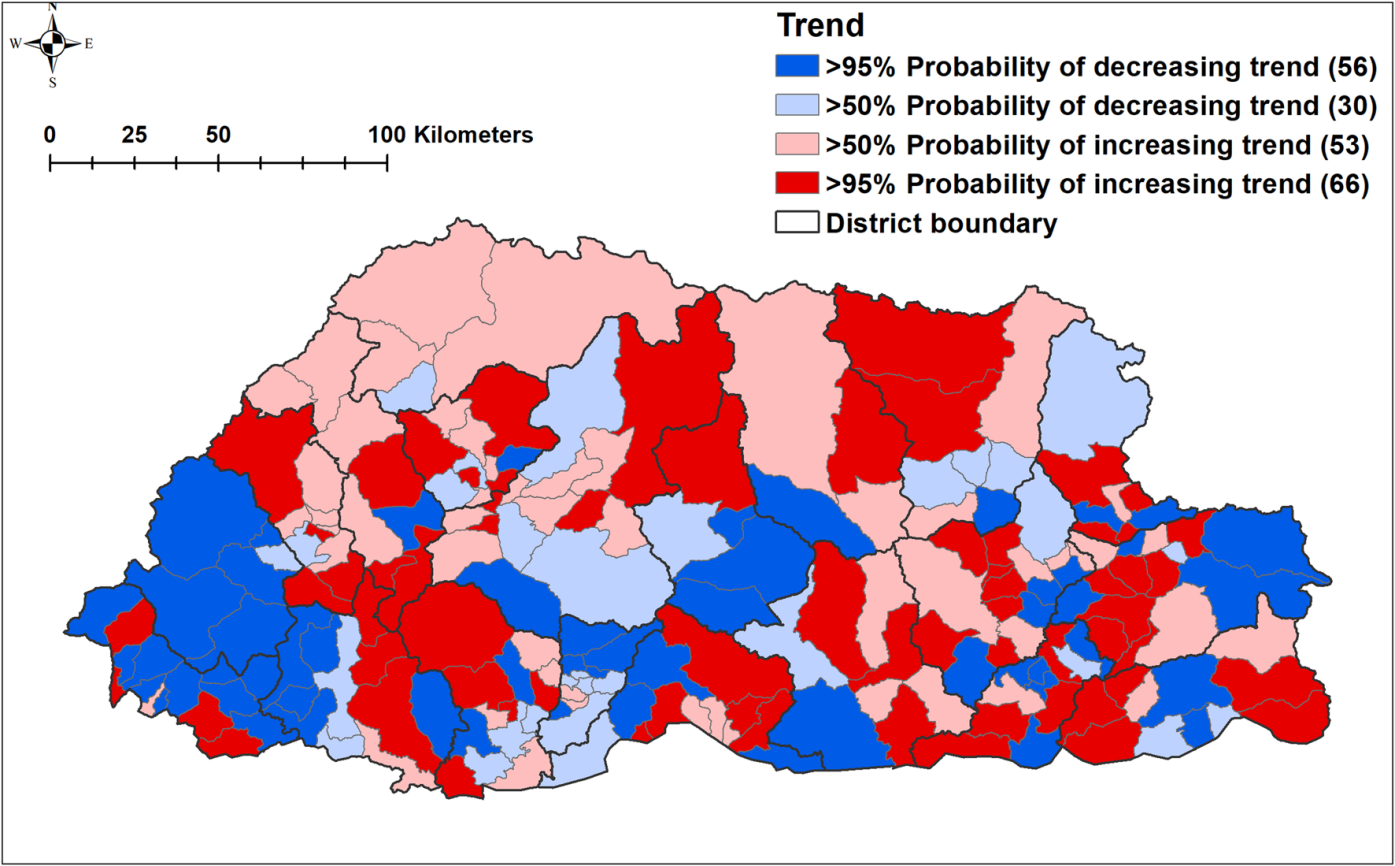
Trend of pneumonia burden by sub-district in Bhutan during the study period, 2010–2018. (Maps were created using ArcMap 10.5 software (ESRI, Redlands, CA).

There was no statistical evidence of spatial clustering after accounting for the covariates (Table 2 and Fig. 4). There was > 95% probability of a lower than the national average trend of pneumonia in 56/205 sub-districts, whereas 66/205 sub-districts had > 95% probability of a trend more than the national average. There was no clear spatial pattern, with sub-districts showing higher and lower average trends across all the 20 districts (Fig. 5).

## Discussion

This is the first study to have explored the climatic and geographic effects on childhood pneumonia in Bhutan. Pneumonia was spatially and temporally heterogeneous across sub-districts of Bhutan during the study period. There was a decreasing trend in disease burden from 2010 through 2018, in addition to a strong seasonal pattern. Pneumonia mainly affected males and children aged < 5 years. Maximum temperature were associated with an increased incidence of pneumonia, while relative humidity was associated with a decreased incidence of disease.

In addition to climatic factors, spatial heterogeneity could be due to differences in the socio-demographic characteristics of sub-districts. Known risk factors responsible for exacerbation and spread of pneumonia in Bhutan include low birth weight, malnutrition, indoor air pollution, overcrowded households, lack of breastfeeding in infants, and poor personal and environmental hygiene^24,25^. This was evident from the two districts of Haa and Paro, which have the lowest poverty levels^26^, and also reported lowest SMR associated with pneumonia. Similar to the decreasing trend in the incidence of global childhood pneumonia^6^, the pneumonia trend in Bhutan decreased during the study period from 2015 onwards. This could be attributed to a decrease in exposure to key risk factors including poor housing conditions and overcrowding, incomplete immunisation and malnutrition^6^.

Pneumonia is the single largest infectious cause of death in children worldwide. It accounts for 15% of all deaths of children < 5 years^27^. In this study, children < 5 years were at a much higher risk of pneumonia compared to those 5–14 years old. Infants (aged between 0–11 months) were reported to contribute up to 24.2% of pneumonia cases in another study from Bhutan^28^. The WHO and United Nations Children’s Fund (UNICEF) initiated a global action plan for pneumonia and diarrhoea (GAPPD) to accelerate pneumonia control in children^29^. The GAPPD strategies include promoting: (1) exclusive breastfeeding and adequate complementary feeding to protect children from pneumonia; (2) vaccination; (3) hand hygiene; (4) reductions in household air pollution; (5) HIV prevention; (6) co-trimoxazole prophylaxis for HIV infected and exposed children; and (7) treatment of childhood pneumonia with antibiotics and oxygen. Strengthening GAPPD strategies should be considered in Bhutan, as is the case in other countries in South Asia (including Bangladesh and India). The introduction of pneumococcal conjugate vaccines (PCVs) in Bhutan in 2019 is timely in prevention of pneumonia, and the effect of introduction of PCV on the burden of childhood pneumonia in Bhutan must be evaluated^21,30^. Exclusive breastfeeding rates from birth until six months in Bhutan varies from 35.9 to 51.0%^31,32^. Increasing exclusive breastfeeding rates are likely to reduce pneumonia associated morbidity^33^.

Pneumonia incidence was highly seasonal, and was associated with climatic factors including temperature and relative humidity. The association of temperature with pneumonia has been reported in other studies^34,35^. A plausible explanation is the association of higher temperature with air pollution^36^ which in itself is known risk factor and cause of pneumonia^7,37–39^. Most industries are located in the southern parts of Bhutan where air pollution is expected to be higher as compared to other districts. This was reflected by these sub-districts having higher SMR for pneumonia. Additionally, traditional methods of cooking in rural Bhutan using fire wood could also contribute to respiratory illness such as pneumonia^40,41^.

The incidence of pneumonia tends to be higher during the rainy season but not so in this study^42–44^. Rainfall may trigger socio-ecological behavioural changes such as increased contact between people and the distribution of pathogens. Further, heavy rainfall during the monsoon is likely to pollute drinking water, particularly surface water from streams, which is the main drinking water source for rural populations^45^. Unsafe drinking water and sanitation are important drivers of pneumonia^46^. Relative humidity was associated with a decrease in pneumonia incidence in this study which is in concordance with other studies^35,47^. Higher relative humidity decreases the survival of lipid-enveloped viruses such as influenza A, influenza b and respiratory syncytial virus^48,49^.

There are a number of limitations that need to be considered when interpreting the results of this study. First, the study used routine case reports to measure pneumonia incidence. Known issues exist surrounding completeness and representativeness of such data. Secondly, the causal organisms of pneumonia were not available and the association could be different based on the organisms. Thirdly, there was no reconciliation to accommodate different levels of aggregation of the climate variables (district) and the disease data (sub-district), and the climate conditions were assumed to be homogeneous within a district. Lastly, unaccounted risk modifiers were not included in the modelling due to a lack of available data. These important unmeasured factors, such as immunization coverage, air pollution level, living standards and socio-economic status, crowding, smoking, access to safe drinking water and latrine usage might have resulted in confounding, which was not able to be quantified^39,50,51^.

Despite these limitations, the strengths of this study are the capacity to implement the spatial analysis at a relatively fine resolution, being the sub-district level, and over an extended time series (108 months). Traditionally, spatial patterns of infectious disease risk have been displayed at larger geographical units, such as a district, province**,** national, regional, and global scales^45,52,53^. Such low resolution can mask localized disease patterns due to averaging^54^.

## Conclusion

Pneumonia is an important childhood disease and the introduction of PCVs to reduce the burden of this disease is timely. Pneumonia was highly seasonal and spatially heterogeneous across sub-districts. Seasonality can be explained by climatic factors including maximum temperature and relative humidity. The spatial and temporal variability of pneumonia should inform prevention and control efforts in Bhutan, through rational decision making and proper resources allocation.

## Materials and methods

### Study area

Bhutan, located in the Eastern Himalayas, borders China in the north and India in the east, south and west. The country is divided administratively into 20 districts and 205 sub-districts, with a total projected population of 735,553 (189,446 under 15 years) in 2017^55^. Around 62.2% (452,178) of the population live in rural areas and practice subsistence farming. The altitude ranges from 75 m above sea level in the south to more than 7000 m in the Himalayas (Fig. 1).

### Study design and data source

This is a retrospective study using secondary data on pneumonia from January 2010 to December 2018, stratified by sex and age (< 5 years and 5–14 years) at the sub-district level. The data were obtained from the National Acute Respiratory Infections surveillance system, hosted by the Bhutan Health Information and Management Systems (HIMS) under the Bhutan Ministry of Health. These data contain all pneumonia cases treated by health centres including hospitals and primary health care facilities and reported to the HIMS every month. Pneumonia is defined as “a patient with history of cough or reported breathing difficulty, and increased respiratory rate, (respiratory rate ≥ 50 breaths per minute in children aged two months or more and less than 12 months, or repiratory rate ≥ 40 breaths per minute in children aged 12 months or more and less than 60 months) or chest indrawing”^56^. Daily climatic variables (rainfall, relative humidity, minimum and maximum temperature) were obtained from the National Centre for Hydrology and Meteorology under the Ministry of Economic Affairs of Bhutan. Monthly average climatic variables were calculated at the district level because they were available only at the district level. Population estimates used in the study were obtained from the National Statistical Bureau, Bhutan^57^. Administrative boundary maps were downloaded from the DIVA-GIS website^58^.

### Crude standardized morbidity ratios

An initial descriptive analysis of pneumonia incidence across the country was conducted. Crude SMR for each sub-district were calculated using the following formula:

$${Y}_{i}= \frac{{O}_{i}}{{E}_{i}}$$

where *Y* is the overall SMR in sub-district *i, O* is the total number of observed pneumonia cases over the entire study period in the sub-district and *E* is the expected number of pneumonia cases in the sub-district across the study period. The expected number was calculated by multiplying the national incidence by the average population for each sub-district over the study period.

### Exploration of seasonal patterns and inter-annual patterns

The time series of pneumonia incidence was decomposed using STL weighted regression to show: the seasonal pattern, inter-annual patterns and the residual variability. The STL model was structured as follows:

$${Y}_{t}={S}_{t}+{T}_{t}+{R}_{t}$$

where *Y*_*t*_ represents numbers of local pneumonia cases with logarithmic transformation, *S*_*t*_ is the additive seasonal component, *T*_*t*_ is the trend, and *R*_*t*_ is the “remainder component”; *t* is time in months^59,60^.

### Spatio-temporal model

A Bayesian statistical framework was deployed for spatial analysis. It provides a convenient framework for the simultaneous inclusion of covariates and spatial autocorrelation in a single model, while providing robust evaluation and expression of uncertainty. The posterior distributions can be used to quantify uncertainties in parameters of interest (e.g., covariate effects and spatial patterns of disease risk)^61^.

Initially, a preliminary bivariate Poisson regression of pneumonia cases was undertaken to select the covariates. The covariates were altitude and climatic variables including rainfall, minimum and maximum temperature and relative humidity without lag, and lagged up to 3 months. The covariates with a *p*-value of < 0.05 and the lowest Akaike's information criterion (AIC) were selected (Supplementary Table 1). The co-linearity of the selected climatic and environmental variables was tested using variance inflation factors (VIF) (Supplementary Table 2). In the final model, altitude, and rainfall, maximum temperature without lag and relative humidity lagged at 3 months were included.

Of the 83,315 observations stratified by sub-districts, age (< 5 and 5–14 years) and sex over an observation period of 108 months, there were 63,742 (72%) zero counts of pneumonia. Therefore, Zero-inflated Poisson (ZIP) regression was constructed in a Bayesian framework (Supplementary Table 3). The first model (Model I), assumed that spatial autocorrelation was not present in the relative risk of pneumonia. This model was developed with selected climatic factors (rainfall, maximum temperature and relative humidity), altitude, age (< 5 and 5–14 years) and gender as explanatory variables, and an unstructured random effect for sub-districts. The second model (Model II) contained a spatially structured random effect in addition to the aforementioned covariates. Model III, a convolution model, contained all of the components of the preceding two models. The model with the lowest DIC was selected as the final explanatory model.

Model III assumed that the observed counts of pneumonia, *Y*, for the *i*^th^ sub-district (*i* = 1 … 205) in the *j*^th^ month (January 2010–December 2018) followed a Poisson distribution with mean (μ_*ij*_), that is,

$$P({Y}_{ij}= {y}_{ij})=\left\{\begin{array}{c}\omega +1 \left(1-\omega \right){e}^{-\mu }, {y}_{ij}=0\\ \left(1-\omega \right){e}^{-\mu } {\mu }_{ij}^{{y}_{ij}}/{y}_{ij}, {y}_{ij}>0;\end{array}\right.$$

$$Y_{ij} \sim {\text{Poisson}}\left( {\mu_{ij} } \right)$$

$${\text{log}}\left( {\mu_{ij} } \right) = {\text{log}}\left( {{\text{E}}_{ij} } \right) + \theta_{ij}$$

$$\theta_{ij} = \alpha + \beta_{1} \times {\text{Age }} + \beta_{2} \times {\text{Sex}} + \beta_{3} \times {\text{trend}}_{j} + \beta_{4} \times {\text{Altitude}} + \beta_{5} \times {\text{Rainfall}}_{ij} + \beta_{6} \times {\text{Humidity}}_{ij} + \beta_{7} \times {\text{Tempmax}}_{ij} + {\text{u}}_{i} + {\text{s}}_{i} + {\text{w}}_{i}$$

where the expected number of cases in sub-district *i*, month *j* (acting as an offset to control for population size) was represented by E_*ij*_ and *θ*_*ij*_ is the mean log relative risk (RR). The intercept (α), and coefficients for age (5–14 years as reference), sex (male as reference), monthly trend, altitude, rainfall, relative humidity and maximum temperature are *β*_*1*_*, β*_*2*_*, β*_*3*_*, β*_*4*_*, β*_*5*_*, β*_*6*_ and *β*_*7*_*.* The spatially unstructured and structured random effects are represented as u_*i*_ and s_*i*_, respectively, with u_*i*_ excluded from Model II and s_*i*_ excluded from Model I. Spatiotemporal random effect with a mean of zero and variance of σ_w_^2^ was denoted by w_*i*_ as in other studies^62,63^.

A conditional autoregressive (CAR) prior structure was used to model the spatially structured random effect. Spatial relationships between the sub-districts were based on a ‘queen’ contiguity matrix^64^. A weight of 1 was assigned to sub-districts sharing a border and 0 otherwise. A flat prior distribution was specified for the intercept, whereas non-informative normal prior distributions (mean = 0.0, precision = 0.00001) were used for the coefficients. The priors for the precision of unstructured and spatially structured random effects were specified using non-informative gamma distributions with shape and scale parameters of 0.5, 0.001.

Markov Chain Monte Carlo simulation was used to estimate model parameters^65^. The models were run for an initial 10,000 iterations for burn in, which were then discarded. Subsequently, visual inspection of posterior density and history plots were used to note convergence at intervals of 20,000 iterations. Convergence occurred at approximately 100,000 iterations for all models. Following convergence, posterior distributions from model parameters were stored for inference. Summaries of parameters were calculated, including posterior mean and 95% CrI. In the Bayesian analyses, RR with 95% CrI that excluded 1 were considered statistically significant.

Seasonality decomposition was carried out using the R statistical software, version 3.3.1^66^. The ZIP regression model was constructed using WinBUGS software, version 1.4.3 (MRC Biostatistics Unit 2008)^67^. ArcMap 10.5 software (ESRI, Redlands, CA) was used to generate maps of the posterior means of the unstructured and structured random effects and the spatiotemporal random effects.

### Ethical approval and patient confidentiality

Administrative approval to use these datasets was provided by the Ministry of Health, Bhutan. This study was a low-risk study since the surveillance data did not contain identifying information on individual participants.

## Supplementary Information

Supplementary Information.

## Data Availability

The datasets of the current study will be made available from the corresponding author on reasonable request.
