# Author Correction: Spatio-temporal patterns of childhood pneumonia in Bhutan: a Bayesian analysis

**DOI:** 10.1038/s41598-021-01075-y

**Published:** 2021-11-15

**Authors:** Kinley Wangdi, Kinley Penjor, Tsheten Tsheten, Chachu Tshering, Peter Gething, Darren J. Gray, Archie C. A. Clements

**Affiliations:** 1 Department of Global Health, Research School of Population Health, College of Health and Medicine, Australian National University, Canberra, Australia; 2 Vector-Borne Diseases Control Programme, Department of Public Health, Ministry of Health, Thimphu, Bhutan; 3 Royal Centre for Disease Control, Ministry of Health, Thimphu, Bhutan; 4 Child Health Program, Communicable Diseases Division, Department of Public Health, Ministry of Health, Thimphu, Bhutan; 5 Telethon Kids Institute, Nedlands, Australia; 6 Faculty of Health Sciences, Curtin University, Perth, Australia

Correction to: *Scientific Reports* 10.1038/s41598-021-99137-8, published online 14 October 2021

The original version of this Article contained an error in Figure 3 where the crude standardized morbidity ratios were incorrectly calculated, resulting in errors in the map and key. The original Figure 3 and accompanying legend appear below.

**Figure 3:**
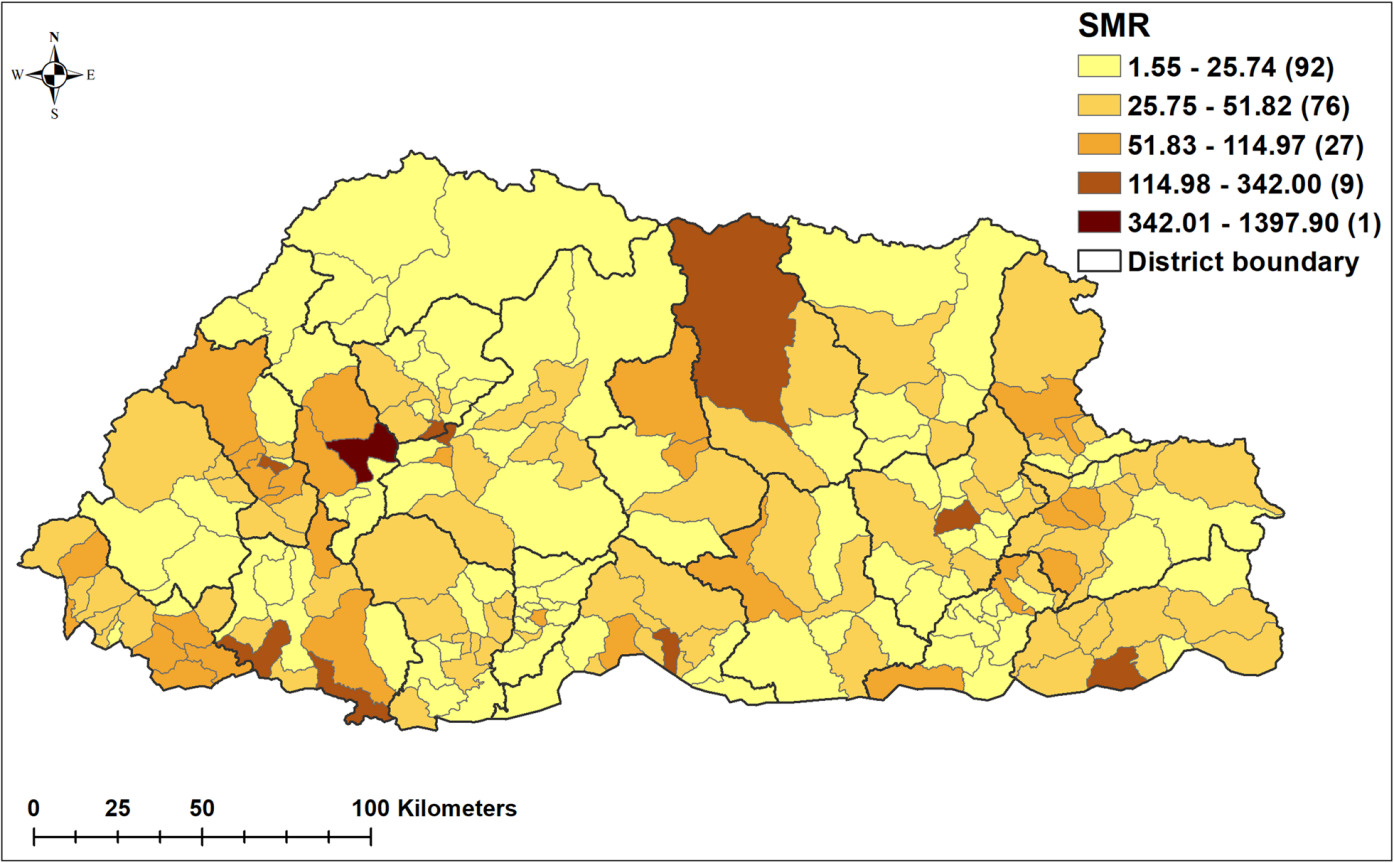
Crude standardized morbidity ratios (SMR) of pneumonia by sub-district during the study period, 2010–2018. (Maps were created using ArcMap 10.5 software (ESRI, Redlands, CA).

As a result, in the Results section under the subheading ‘Descriptive analysis’,

“The average standardized morbidity ratio (SMR) of pneumonia at sub-district level was 44.72 (range: 1.55 to 1397.9; Standard Deviation of 102.05).”

now reads:

“The average standardized morbidity ratio (SMR) of pneumonia at sub-district level was 9.6 (range: 0 to 49.8; Standard Deviation of 9.7).”

Additionally,

“The SMR for Haa, Paro, Gasa, Bumthang and Wandue was lower than average, whilst for Bumthang, Chukha, Mongar, Samtse, Sarpang, Samdrup Jongkhar and Thimphu districts, the SMR was higher than average (Fig. 3).”

now reads:

“The SMR for Haa, Paro, Gasa, Bumthang and Wandue was lower than average, whilst for Chukha, Mongar, Samtse, Sarpang, Samdrup Jongkhar and Thimphu districts, the SMR was higher than average (Fig. 3).”

The original Article has been corrected.

