# Supplementary material for: Spatio-temporal patterns of childhood pneumonia in Bhutan: a Bayesian analysis
Source: Sci Rep. 2021 Oct 14;11:20422. doi: 10.1038/s41598-021-99137-8 (PMC8516968; doi:10.1038/s41598-021-99137-8)
Supplement: Supplementary file 1 — Supplementary Information. [file 41598_2021_99137_MOESM1_ESM.pdf]

Spatio-temporal patterns of pneumonia in Bhutan: A Bayesian analysis

Kinley Wangdi[1†*], Kinley Penjor[2*], Tsheten Tsheten[1,3], Chachu Tshering[4], Peter Gething[5,6], Darren J Gray[1], Archie CA Clements[5,6]

[1]Department of Global Health, Research School of Population Health, Australian National University, Canberra, Australia

[2]Vector-borne Diseases Control Programme, Department of Public Health, Ministry of Health, Bhutan

[3]Royal Centre for Disease Control, Ministry of Health, Bhutan

[4] Child Health Program, Communicable Diseases Division, Department of Public Health

Ministry of Health, Thimphu, Bhutan

[5]Telethon Kids Institute, Nedlands, Australia

[6]Faculty of Health Sciences, Curtin University, Perth, Australia

[†]Corresponding author

[*]Co-first author

Authors:
KW: kinley.wangdi@anu.edu.au

KP: kinleyp@health.gov.bt

TT: tsheten.tsheten@anu.edu.au

CT: ctshering@health.gov.bt

PG: peter.gething@telethonkids.org.au

DG: darren.gray@anu.edu.au

ACAC: archie.clements@curtin.edu.au

Supplementary Tables

Supplementary Table 1 Poisson regression for the selection of most significant variables

**Variables**	**IRR**	**95% CI**	**p value**	**AIC**	**BIC**
**Without lag**					
Altitude	0.9996	0.99956 .9997	<0.0001	229381.9	229397.8
Rainfall	1.02	1.022 - 1.023	<0.0001	**239354.5**	**239370.5**
Max Temperature	1.07	1.065-1.068	<0.0001	**235617.4**	**235633.4**
Min Temperature	1.02	1.016 - 1.017	<0.0001	**233902.7**	**233918.7**
Relative humidity	0.99	0.99 - 0.9904	<0.0001	240493.7	240509.7
**One month lag**					
Rainfall	1.02	1.021 - 1.022	<0.0001	239684.2	239700.3
Max Temperature	1.06	1.055 - 1.058	<0.0001	237938.9	237954.9
Min Temperature	1.02	1.015 - 1.016	<0.0001	234721.9	234737.9
Relative humidity	0.99	0.989 - 0.99	<0.0001	240035.3	240051.3
**Two months lag**					
Rainfall	1.02	1.018 - 1.019	<0.0001	241115.2	241131.2
Max Temperature	1.04	1.040 - 1.043	<0.0001	240727.3	240743.3
Min Temperature	1.02	1.014 - 1.015	<0.0001	236067.3	236083.3
Relative humidity	0.99	0.988 - 0.989	<0.0001	238672.4	238688.4
**Three months lag**					
Rainfall	1.01	1.012 - 1.014	<0.0001	242911.1	242927.1
Max Temperature	1.03	1.023 - 1.026	<0.0001	242969.0	242985.1
Min Temperature	1.01	1.013 - 1.014	<0.0001	237640.1	237656.2
Relative humidity	0.99	0.987 - 0.988	<0.0001	**237310.1**	**237326.1**

Supplementary Table 2 Collinearity of selected climatic and environmental variables using variance inflation factors (VIF)

**Variable**	**VIF**	**1/VIF**
Rainfall	1.34	0.744835
Maximum Temperature	1.52	0.656894
Minimum Temperature	3.88	0.257439
Relative humidity lagged 3 months	2.68	0.373719

Altitude	1.23	0.812218
**Mean VIF**	**2.13**	

Supplementary Table 3 Model comparison using Akaike's information criterion and Bayesian information criterion

Models	Observations	AIC	BIC
**Poisson**	21,816	221751.1	221791.1
**ZIP**	21,816	161437.0	161485.0

Supplementary Figures

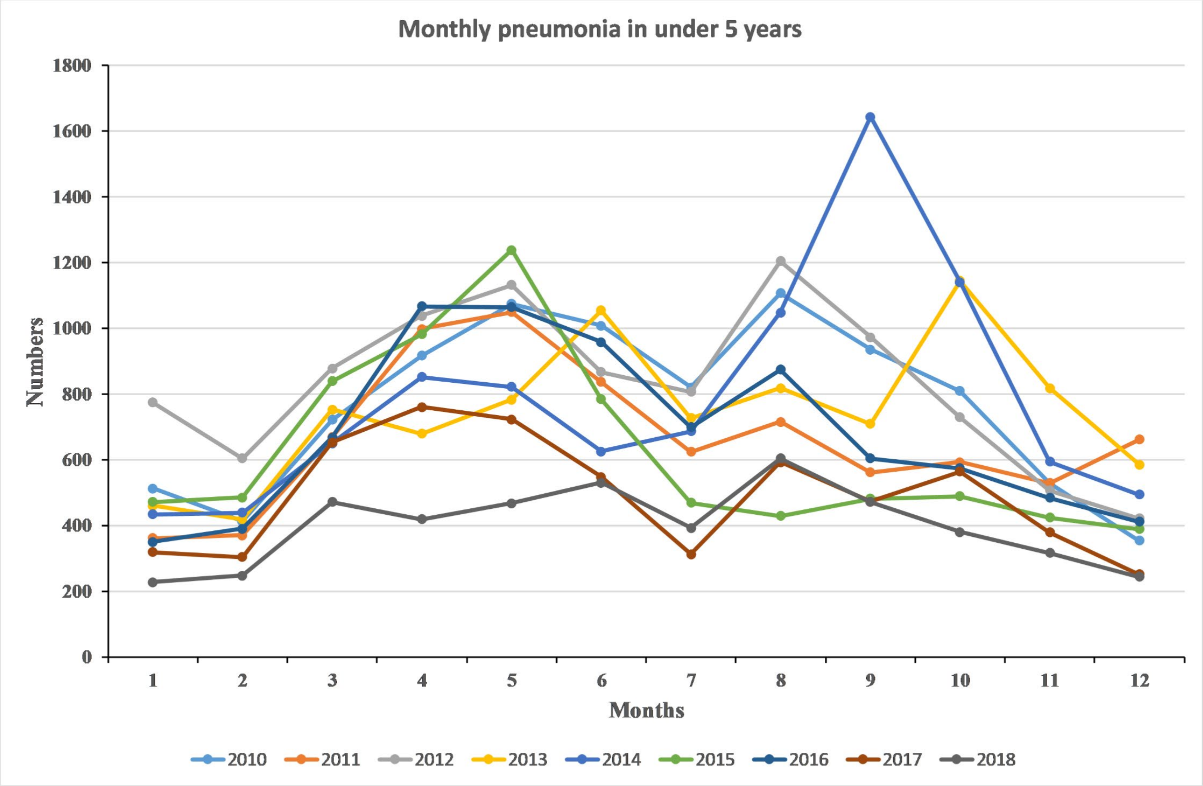

Supplementary Fig. 1 Monthly trend of pneumonia in under 5 years.

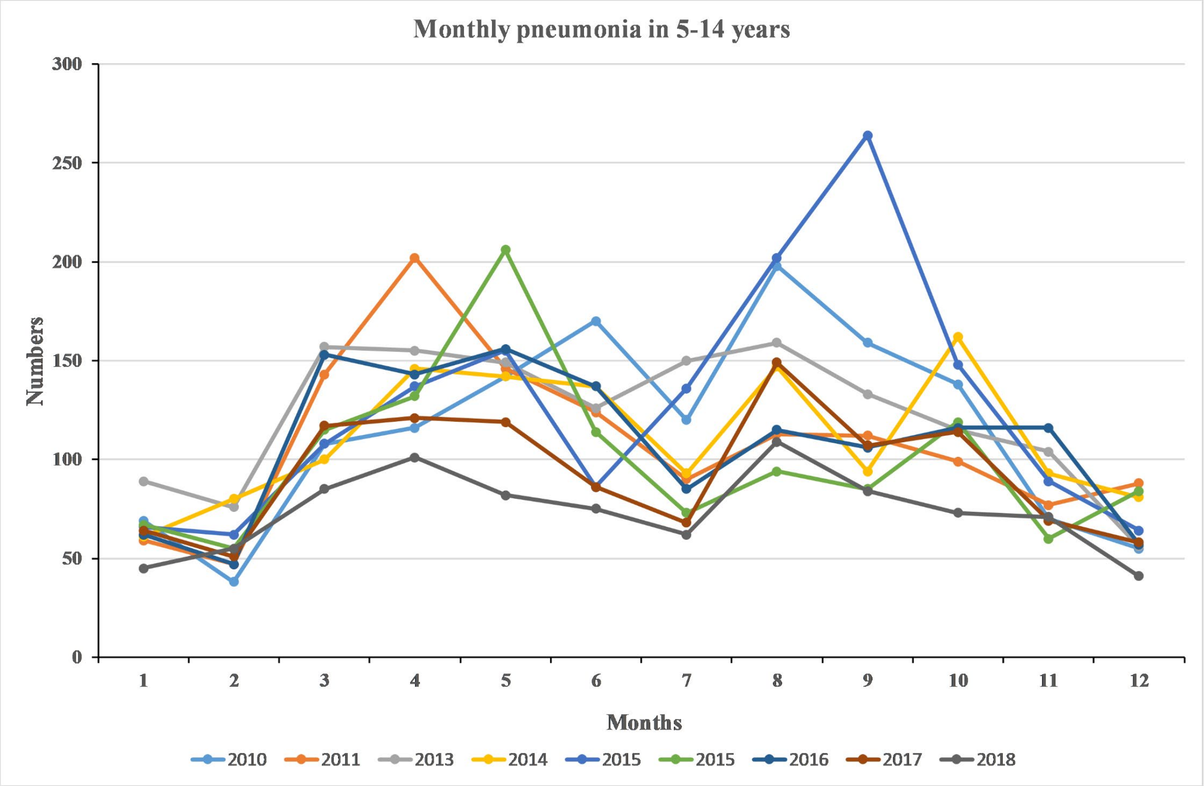

Supplementary Fig. 2 Monthly trend of pneumonia in 5-14 years.
